# Determination of antibiotic resistance patterns and genotypes of *Escherichia coli *isolated from wild birds

**DOI:** 10.1186/s40168-023-01729-1

**Published:** 2024-01-08

**Authors:** Nejash A. Ahmed, Timur Gulhan

**Affiliations:** 1 Department of Veterinary Microbiology, Faculty of Veterinary Medicine, Ondokuz Mayis University, Samsun, Turkey; 2 Daro Lebu District Agriculture Office, Mechara-Micheta, Ethiopia

**Keywords:** Antibiotic resistance, *E. coli*, Gull, Pigeon, RAPD-PCR

## Abstract

**Background:**

Curbing the potential negative impact of antibiotic resistance, one of our era's growing global public health crises, requires regular monitoring of the resistance situations, including the reservoir of resistance genes. Wild birds, a possible bioindicator of antibiotic resistance, have been suggested to play a role in the dissemination of antibiotic-resistant bacteria. Therefore, this study was conducted with the objective of determining the phenotypic and genotypic antibiotic resistance profiles of 100 *Escherichia coli* isolates of gull and pigeon origin by using the Kirby-Bauer disk diffusion method and PCR. Furthermore, the genetic relationships of the isolates were determined by RAPD-PCR.

**Results:**

Phenotypic antibiotic susceptibility testing revealed that 63% (63/100) and 29% (29/100) of *E. coli* isolates were resistant to at least one antibiotic and multidrug-resistant (MDR), respectively. With the exception of cephalothin, to which the *E. coli* isolates were 100% susceptible, tetracycline (52%), kanamycin (38%), streptomycin (37%), ampicillin (28%), chloramphenicol (21%), trimethoprim/sulfamethoxazole (19%), gentamicin (13%), enrofloxacin (12%) and ciprofloxacin (12%) resistances were detected at varying degrees. Among the investigated resistance genes, *tet(B)* (66%), *tet(A)* (63%), *aphA1* (48%), *sul3* (34%), *sul2* (26%), *strA/strB* (24%) and *sul1* (16%) were detected. Regarding the genetic diversity of the isolates, the RAPD-PCR-based dendrograms divided both pigeon and gull isolates into five different clusters based on a 70% similarity threshold. Dendrogram analysis revealed 47–100% similarities among pigeon-origin strains and 40–100% similarities among gull-origin *E.coli* strains.

**Conclusions:**

This study revealed that gulls and pigeons carry MDR *E. coli* isolates, which may pose a risk to animal and human health by contaminating the environment with their feces. However, a large-scale epidemiological study investigating the genetic relationship of the strains from a "one health" point of view is warranted to determine the possible transmission patterns of antibiotic-resistant bacteria between wild birds, the environment, humans, and other hosts.

Video Abstract

**Supplementary Information:**

The online version contains supplementary material available at 10.1186/s40168-023-01729-1.

## Background

Wild animals, including wild birds, are considered part of our ecosystem’s biodiversity, contributing to the continuity of the normal ecosystem on Earth. Assessing their possible epidemiological role as a carrier of infection contributes to the assessment of ecosystem health status as well as diseases that can affect other hosts, including humans [1]. It has been proposed that wild birds may act as reservoirs of pathogenic microorganisms [1–3], suggesting that the diseases carried by wild birds may affect the health of other potential hosts [4–6].

*Escherichia coli* (*E. coli*), *Campylobacter,* and *Salmonella*, common zoonotic bacterial pathogens that cause severe human infections [7], are among the most frequently reported pathogens associated with wild birds [4, 8]. Even though *E. coli* is a commensal pathogen in the intestines of humans and different animal species, including avian species, it is one of the most common animal and human pathogens responsible for significant infections [9]. Moreover, this bacterium is known to transfer its resistance genes to other bacteria besides acquiring resistance genes from other bacteria [10]. Hence, this bacterium is used as a model bacteria for detecting antibiotic resistance in veterinary and human medicine [11].

Currently, antimicrobial-resistant microorganisms have become one of the biggest health challenges in public health and veterinary medicine [12–14]. This growing threat of antibiotic resistance can significantly impact both sectors by limiting antibiotic options [15]. One critical factor that aggravates this situation is the improper use of antibiotics [16]. Several genes conferring resistance to drugs, which are highly important in human and animal health, have been reported in *E. coli* isolated from wild birds from different parts of the world [17–19], showing the commonness of antibiotic-resistant pathogens originating from other hosts (e.g., animals and humans) in the environment [20]. Therefore, wild birds, which are not directly exposed to antibiotic agents, could become infected by resistant bacteria through contact with a contaminated environment (for example, contaminated water/food) [21, 22]. In support of this argument, molecular studies have reported the genetic similarities of *E. coli* strains obtained from wild birds, landfills, wastewater, and humans [23, 24]. The carriage of drug-resistant *E. coli* isolates by wild birds suggests that wild birds may serve as a reservoir in the transmission of this pathogen to domestic animals and also cause environmental contamination [1, 24].

In general, several studies have highlighted the possible role of wild birds as the carriers/reservoirs of antibiotic-resistant *E. coli* pathotypes [3, 22, 25–28] although the situation in developing countries is not adequately elucidated yet. In Turkey, despite the availability of some studies [5, 29, 30] on isolation and antibiotic susceptibility testing, antibiotic resistance patterns and the resistance genes' distribution in wild bird populations have not been clearly elucidated. However, addressing the growing challenge of antibiotic resistance requires a detailed understanding of potential sources and vectors of resistance genes [31]. Hence, determining antibiotic resistance patterns and genes that cause resistance in bacteria obtained from hosts like wild birds for which sufficient data are unavailable is of great importance in designing effective prevention strategies against antibiotic resistance challenges. Therefore, this study aimed to determine the phenotypic and genotypic antibiotic resistance profile of *E. coli* isolated from wild bird species in close contact with humans (gulls and pigeons). Moreover, the isolates’ genetic diversity was analyzed by using RAPD-PCR.

## Material and method

### Bacterial isolates

The bacterial isolates used in this study were obtained from culture collections of the Department of Veterinary Microbiology, Ondokuz Mayis University, Turkey. The isolates were comprised of 100 *E. coli* isolates of gulls (50) and pigeons (50) origin, which were stored at − 20 °C. The isolates obtained from culture collections were isolated from fecal sample of healthy wild birds collected in different times from the Black Sea Region of Turkey. MacConkey agar, eosin methylene blue (EMB), and tryptic soy agar (TSA) were used for the revival and purification of the isolates. *E. coli* isolates were first inoculated on MacConkey agar (Oxoid, UK), and then a single colony was taken from MacConkey agar and inoculated on EMB (Oxoid, UK) agar. Similarly, a colony grown on the EMB agar was taken and sub-cultured on TSA (Oxoid, UK) to be used for various purposes (e.g., DNA extraction). All media were incubated at 37 °C for 24 h to allow bacterial growth.

### DNA extraction

In order to obtain the bacterial DNA, a few pure cultured colonies grown on TSA were selected and suspended in an Eppendorf tube containing 500 µl of sterile distilled water. The mixture was then vortexed and boiled at 100 °C for 10 min and centrifuged at 10,000 rpm for 10 min. The supernatant obtained from the centrifugation process was stored at − 20 °C to be used as the template DNA [3, 32].

### E. coli genotypic confirmation

PCR-based genotypic confirmation of *E. coli* isolates was performed by modifying and optimizing the protocol previously described by Abd El-Razik et al. [33]. For this purpose, 16S rRNA primer pair (Eco 2083 (F): 5'-GCTTGACACTGAACATTGAG-3′; Eco 2745 (R): 5'-GCACTTATCTCTTCCGCATT-3′) were used, and 662 bp bands were considered positive. A PCR mixture comprised of a total volume of 25 μl mixtures which include 10 × PCR buffer (2.5 µl), MgCl2 (3 µl), primer (0.25 µl for each primer), deoxynucleotide triphosphate (dNTP) (0.5 µl), *Taq* polymerase (0.3 µl), 13.2 μl of sterile distilled water, and template DNA (5 μl). The mixture prepared according to the determined protocol was subjected to amplification conditions consisting of initial denaturation for 2 min at 95 °C, followed by 35 cycles of denaturation (45 s at 94 °C), annealing (45 s at 57 °C), and extension (45 s at 72 °C), and final extension for 10 min at 72 °C. After mixing the amplified PCR product with the loading dye, 10 µL of the mixture was taken using a micropipette and loaded into the wells of agarose gel (1.5%) containing 2 µg/ml ethidium bromide. The mixture was then subjected to gel electrophoresis at 150 V for 60 min and visualized using an ultraviolet (UV) transilluminator. To determine the amplicons’ length and whether the band with targeted amplicon sizes (662 bp) was formed or not, DNA Marker (Thermo Scientific, SM0241, 100 bp DNA Ladder) was used. *E. coli* ATCC® 25,922 strain was used as a positive control, whereas a mixture without target DNA was used as a negative control.

### Antibiotic susceptibility test

The Kirby–Bauer standard disk diffusion method was used to determine the resistance patterns of isolates against commonly used antibiotics following the protocols specified in CLSI guidelines [34–36]. Resistance/susceptibility to ampicillin (AMP; 10 µg), trimethoprim/sulfamethoxazole (SXT; 1.25/23.75 µg), cephalothin (KF; 30 µg), tetracycline (TE; 30 µg), chloramphenicol (C;30 µg), streptomycin (S;10 µg), kanamycin (K;30 µg), enrofloxacin (ENR;5 µg), gentamicin (CN; 10 µg), and ciprofloxacin (CIP;5 µg) was investigated. The selection of antibiotics was done in a way that represent major group of antibiotics (beta-lactams, aminoglycoside, phenicol, fluroquinolones, and folate antagonist) taking in to account their common usage and also their public and animal health importance.

The bacterial suspensions used for determining the resistance pattern were prepared by suspending the colonies grown on TSA in sterile physiological saline and adjusting the turbidity to the 0.5 McFarland standard. The prepared suspensions were spread on Mueller–Hinton agar (MHA) within 15 min. Following this, antibiotic discs were aseptically placed on the MHA surface with the help of sterile forceps and incubated overnight at 37 °C under aerobic conditions. Interpretation of the results was performed according to CLSI criteria by measuring the diameters of the inhibition zones and recorded as resistant (R), moderate (I), or susceptible (S) [34–36]. *E. coli* ATCC® 25,922 strain was used as the quality control strain. Bacterial isolates found resistant to at least three antibiotics in different classes (≥ 3 antibiotic groups) were considered as MDR isolates [15]. However, in analyzing the isolates’ resistance profile, only isolates that showed phenotypic resistance to a particular antibiotic were considered (intermediate resistance isolates were not taken into account).

### Antibiotic resistance genes determination

Simplex and multiplex PCR (mPCR) was employed to determine antibiotic resistance genes using specific primer sets. For this purpose, the antibiotic resistance genes conferring resistance to quinolones, tetracyclines, sulfonamides, and aminoglycosides were investigated by modifying and optimizing the PCR protocols described in previous studies (Tables 1 and 2) [37–39]. Quinolone [*qnr(A)*, *qnr(B)* and *qnr(S)*], tetracyclines resistance genes [*tet(A)*, *tet(B)* and *tet(C)*], aminoglycosides (*strA/strB*, *aphA1*, *aphA2, aadB,* and *aac(3) IV*), and sulfonamides (*sul1*, *sul2* and *sul3*) resistance genes were investigated. *qnr* genes’ positive controls were kindly provided by Assoc. Prof. Dr. Yeliz Tanriverdi Çayci (Ondokuz Mayis University, Faculty of Medicine, Department of Medical Microbiology). For other resistance genes, *E. coli* strains known to have the genes under consideration, obtained from Ondokuz Mayıs University Veterinary Microbiology Laboratory, were used.

**Table 1:** The concentration of PCR components used in determining the antibiotic resistance genes

Target resistance gene	PCR components and concentration used						
	10 × PCR buffer	MgCl2 (mM)	dNTP (mM)	Primer cons. (µM)		*Taq* polymerase (U)	Template DNA (µl)
				Forward	Reverse		
*tet(A)*	1 X	2.5	0.2	0.2	0.2	1.5	10
*tet(B)*	1 X	2.5	0.2	0.2	0.2	1.5	10
*tet(C)*	1 X	2.5	0.2	0.52	0.52	1.5	10
*qnr(A)*	1 X	1.5	0.2	0.1	0.1	1.5	3
*qnr(B)*	1 X	2.5	0.2	0.1	0.1	1.5	3
*qnr(S)*	1 X	1.5	0.2	0.1	0.1	1.5	3
*strA/strB*	1 X	2.5	0.16	1	1	1	5
*aac(3)IV*	1 X	3	0.2	0.2	0.2	1.5	5
*aadB*	1 X	2.5	0.08	0.12	0.12	1	5
*aphA1*	Master mix (12.5 µl)			0.16	0.16	-	5
*aphA2*	1 X	2.5	0.08	0.1	0.1	1	5
*sul1*	1 X	2.5	0.2	0.2	0.2	1.5	10
*sul2*				0.3	0.3		
*sul3*				0.2	0.2

**Table 2:** Primers and PCR conditions used in identifying antibiotic resistance genes

Antibiotic group and gene		Primer	Nucleotide sequence(5′–3′)	Product(bp)	Annealing (ºC)	Reference
Sulfonamide	*sul1*	*sul1*-F	CGGCGTGGGCTACCTGAACG	433	66	[37]
		*sul1*-B	GCCGATCGCGTGAAGTTCCG			
	*sul2*	sulII-L	CGGCATCGTCAACATAACCT	721		
		sulII-R	TGTGCGGATGAAGTCAGCTC			
	*sul3*	sul3-GKa-F	CAACGGAAGTGGGCGTTGTGGA	244		
		sul3-GKa-R	GCTGCACCAATTCGCTGAACG			
Quinolone	*qnr(A)*	QnrAm*-*F	AGAGGATTTCTCACGCCAGG	580	59	[38]
		QnrAm-R	TGCCAGGCACAGATCTTGAC			
	*qnr(B)*	QnrBm-F	GGMATHGAAATTCGCCACTG	264	59	
		QnrBm-R	TTTGCYGYYCGCCAGTCGAA			
	*qnr(s)*	QnrSm-F	GCAAGTTCATTGAACAGGGT	428	55	
		QnrSm-R	TCTAAACCGTCGAGTTCGGCG			
Tetracycline	*tet(A)*	TetA-L	GGCGGTCTTCTTCATCATGC	502	63	[37]
		TetA-R	CGGCAGGCAGAGCAAGTAGA			
	*tet(B)*	TetBGK-F2	CGCCCAGTGCTGTTGTTGTC	173	61	
		TetBGK-R2	CGCGTTGAGAAGCTGAGGTG			
	*tet(C)*	TetC-L	GCTGTAGGCATAGGCTTGGT	888	58	
		TetC-R	GCCGGAAGCGAGAAGAATCA			
Aminoglycoside	*strA/strB*	strA-F	ATGGTGGACCCTAAAACTCT	893	52	[37]
		strB-R	CGTCTAGGATCGAGACAAAG			
	*aac(3)IV*	aac4-L	TGCTGGTCCACAGCTCCTTC	653	55	
		aac4-R	CGGATGCAGGAAGATCAA			
	*aadB*	aadB-L	GAGGAGTTGGACTATGGATT	208	52	
		aadB-R	CTTCATCGGCATAGTAAAAG			
	*aphA1*	aph(3´)-Ia F	ATGGGCTCGCGATAATGTC	634	58	[39]
		aph(3')-Ia R	CTCACCGAGGCAGTTCCAT			
	*aphA2*	aphA2-L	GATTGAACAAGATGGATTGC	347	53	[37]
		aphA2-R	CCATGATGGATACTTTCTCG			

M = A or C; H = A or C or T; Y = C or T

For determining sulfonamide resistance, mPCR was performed in a total volume of 25 µl PCR reaction mixture consisting of PCR components presented in Table 1 and sterile distilled water. Other genes were investigated using simplex PCR, and PCR amplifications were performed using the concentration of PCR components presented in Table 1**,** which were adjusted to a total of 25 µl with sterile distilled water. The amplification conditions for *sul1*, *sul2*, and *sul3* consist of pre-denaturation (15 min at 95 °C) followed by 30 cycles of denaturation (for 60 s at 95 °C), annealing (60 s at 66 °C), and elongation (60 s at 72 °C), and final elongation for 10 min at 72 °C. The amplification conditions for the plasmid-mediated quinolone resistance (PMQR) genes (*qnrA*, *qnrB*, and *qnrS*) were as follows: 5 min of pre-denaturation at 95 °C, 35 cycles of denaturation at 95 °C for 60 s, 60 s of annealing at the specific primer temperature presented in Table 2, and extension at 72 °C for 1 min and 5 min of final elongation at 72 °C. *tet(A), tet(B), tet(C), strA/strB, aac(3)IV, aphA1, aphA2*, and *aadB* were subjected to PCR amplification consisting of pre-denaturation at 94 °C for 15 min, 30 cycles of denaturation at 94 °C for 60 s, annealing for 60 s at the specific primer temperature indicated in Table 2, and elongation at 72 °C for 60 s, and the final elongation at 72 °C for 10 min. Amplification products were subjected to gel electrophoresis (1.5% agarose) containing 2 μg/ml ethidium bromide and visualized using a UV transilluminator.

### Genotyping of the isolates by RAPD-PCR

Determination of the *E. coli* isolates’ phylogenetic relatedness was performed by using “enterobacterial repetitive intergenic consensus-2 (ERIC-2)” primer (5′-AAGTAAGTGACTGGGGTGAGCG-3′) as previously described by Versalovic et al. [40]. The PCR amplification reaction mixture consists of 10 × PCR buffer (2.5 µl), MgCl2 (2.5 µl), dNTP (0.5 µl), ERIC-2 primer (0.8 µl), Taq DNA polymerase (0.2 µl), and template DNA (5 µl), which were adjusted to a total of volume 30 µl reaction mixture with DNase/RNase-free distilled water. The amplification conditions for RAPD-PCR consisted of 5 min initial denaturation at 94 °C, 40 cycles of 1 min denaturation at 94 °C, 60 s of annealing at 36 °C, and 3 min extension at 72 °C, and a final extension cycle at 72 °C for 7 min. For visualizing the amplicons, a 1.5% agarose gel was prepared in a 1XTBE buffer, and the amplified products were subjected to gel electrophoresis containing 2 μg/ml ethidium bromide for 80 min at 140 V. The bands formed after electrophoresis were visualized using a UV transilluminator, and the bands formed were recorded. The bands were then analyzed with the image analysis program (Quantity one, BioRad) using the UPGMA (“unweighted pair group method with arithmetic mean”) method and dendrograms were drawn.

### Statistical analysis

Descriptive analysis of data obtained from laboratory results was performed using Microsoft Excel® and SPSS® version 26. A chi-square test (*χ*^2^) was used to determine the difference between gulls and pigeons in terms of carrying *E. coli* isolates that were resistant to at least one antibiotic, two or more antibiotics, multidrug-resistant, and also antibiotic-resistant genes carriage. Moreover, the chi-square test was used to determine the associations between specific antibiotic resistance gene carriage and phenotypic antibiotic resistance expression. The difference/relationship was considered statistically significant when the *p*-value was less than 0.05.

## Results

### Genotypic confirmation of E. coli

PCR-based genotypic confirmation of the isolates revealed 662 bp bands; all were confirmed to be *E. coli*.

### Antibiotic susceptibility test

Disk diffusion-based phenotypic antibiotic susceptibility test revealed that 63% (63/100) of *E. coli* isolates were resistant to at least one antibiotic (≥ 1 antibiotic), and 29% (29/100) were resistant to three or more antibiotic groups (MDR). Out of 100 *E. coli* isolates, 52 (52%), 38 (38%), 37 (37%), 28 (28%), 21 (21%), 19 (19%), and 13 (13%) isolates were found to be resistant to tetracycline, kanamycin, streptomycin, ampicillin, chloramphenicol, trimethoprim/sulfamethoxazole, and gentamicin, respectively. Moreover, 12% of the isolates were resistant to enrofloxacin and ciprofloxacin. However, all isolates (100%) were found to be susceptible to cephalothin.

**Fig. 1:**
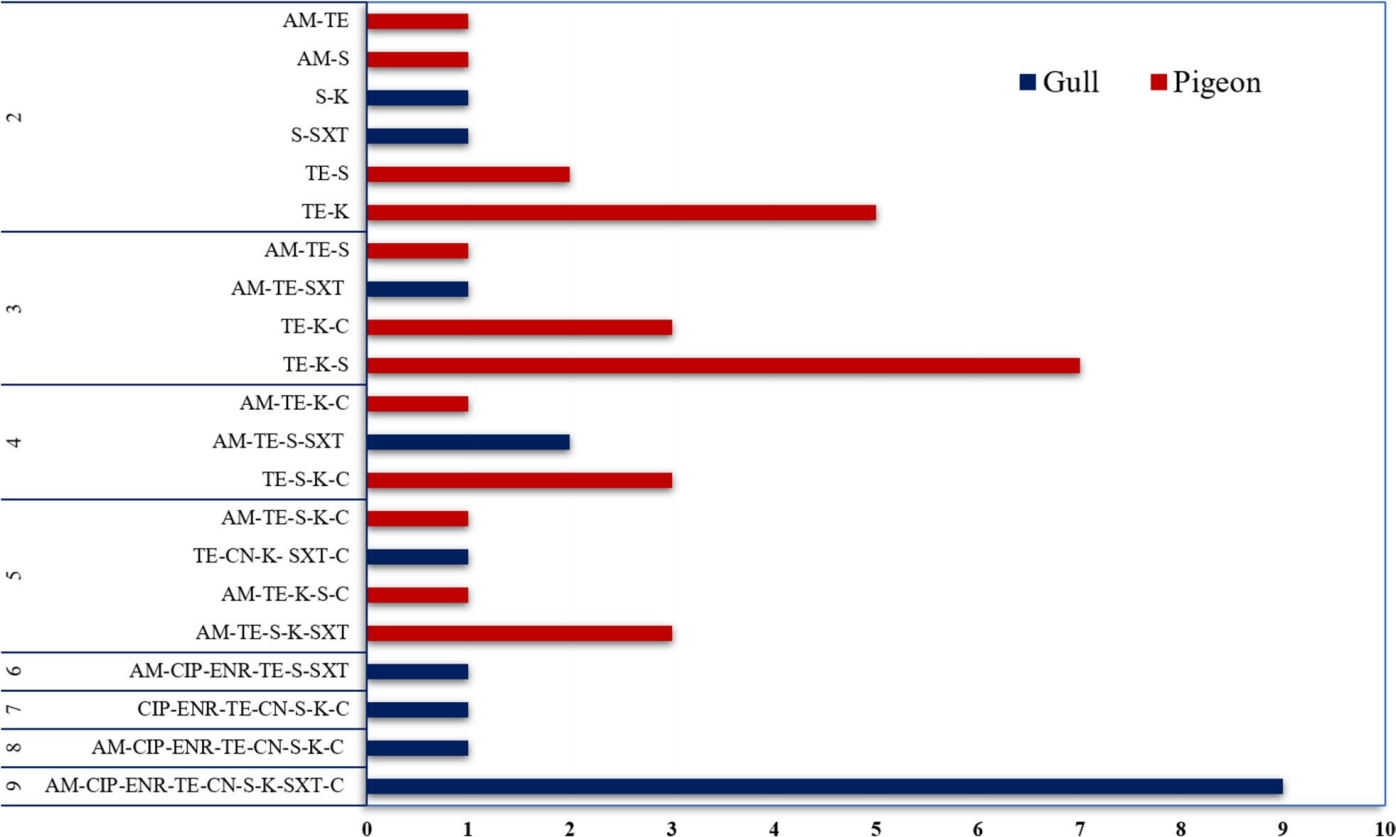
Resistance profile of *E. coli* isolates obtained from gulls and pigeons to multiple antibiotic agents (AM ampicillin, ENR enrofloxacin, CN gentamicin, K kanamycin, C chloramphenicol, CIP ciprofloxacin, S streptomycin, CF cephalothin, TE tetracycline, SXT trimethoprim/sulfamethoxazole)

Looking at the bird species-based results, in pigeon origin isolates, tetracycline (72%) and kanamycin (48%) resistance were comparatively higher, whereas, in gull origin isolates, streptomycin (34%) and trimethoprim/sulfamethoxazole (32%) resistance was found to be relatively higher (Table 3). In terms of carrying isolates that were resistant to at least one antibiotic, a statistically significant (*p* < 0.001, *χ*^2^ = 18.9) difference was found between pigeons (42/50) and gull (21/50) origin isolates. However, in terms of carriage of multidrug-resistant isolates, a statistically non-significant (*p* = 0.50, *χ*^2^ = 0.43) higher MDR rate was found in gull isolates (16/50) than in pigeon isolates (13/50). Nine gull-origin isolates showed resistance to all antibiotics tested except cephalothin. The tetracycline, kanamycin, and streptomycin were detected in seven isolates of pigeon origin (Fig. 1).

**Table 3:** Bird species-based phenotypic antibiotic resistance profile of *E. coli* isolates obtained from gulls and pigeons

Antibiotic		Gull (*n* = 50)			Pigeon (*n* = 50)		
		R (%)	I (%)	S (%)	R (%)	I (%)	S (%)
Beta-lactam	AM	14 (28)	4 (8)	32 (64)	14 (28)	0 (0)	36 (72)
	KF	0 (0)	0 (0)	50 (100)	0 (0)	0 (0)	50 (100)
Fluoroquinolone	CIP	12 (24)	5 (10)	33 (66)	0 (0)	4 (8)	46 (92)
	ENR	12 (24)	4 (8)	34 (68)	0 (0)	0 (0)	50 (100)
Tetracycline	TE	16 (32)	0 (0)	34 (68)	36 (72)	1 (2)	13 (26)
Aminoglycoside	CN	13 (26)	2 (4)	35 (70)	0 (0)	3 (6)	47 (94)
	S	17 (34)	4 (8)	29 (58)	20 (40)	16 (32)	14 (28)
	K	14 (28)	20 (36)	16 (32)	24 (48)	10 (20)	16 (32)
Folate antagonist	SXT	16 (32)	1 (2)	33 (66)	3 (6)	0 (0)	47 (94)
phenicol	C	12 (24)	1 (2)	37 (74)	9 (18)	10 (20)	31 (62)

*AM* Ampicillin, *ENR* Enrofloxacin, *CN* Gentamicin, *K* Kanamycin, *C* Chloramphenicol, *CIP* Ciprofloxacin, *S* Streptomycin, *CF* Cephalothin, *TE* Tetracycline, *SXT* Trimethoprim/sulfamethoxazole

### Antibiotic resistance genes

**Fig. 2:**
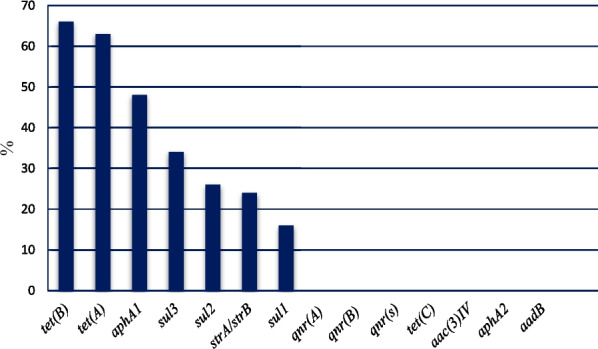
Genotypic antibiotic resistance profile of *E. coli* isolates obtained gulls and pigeons

**Fig. 3:**
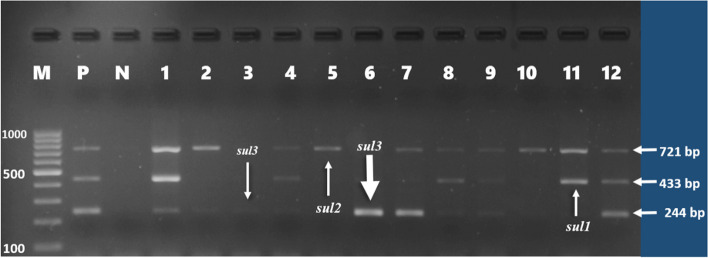
Identification of *sul1* (433 bp), *sul2* (721 bp), and *sul3* (244 bp) genes causing sulfonamide resistance by mPCR (M, marker (Thermo Scientific, SM0241, 100 bp DNA Ladder); P, positive control; N, negative control; 1, 4, 8, 9, 12, *sul1*, *sul2,* and *sul3*), 2 and 7, *sul* 2 and *sul 3*; 3 and 6, *sul3*; 5 and 10, *sul2; 11, sul1* and *sul2*)

**Fig. 4:**
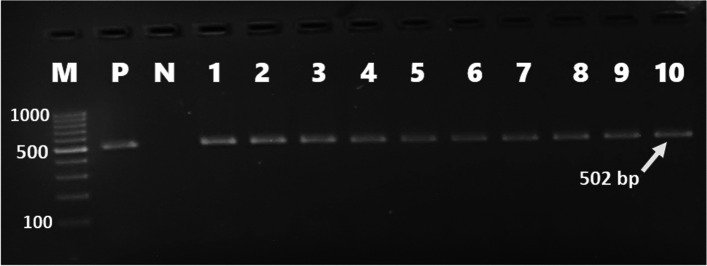
*tet(A)* gene detected using simplex PCR (M marker (Thermo Scientific, SM0241, 100 bp DNA Ladder), P positive control, N negative control, 1–10 positive isolates)

**Fig. 5:**
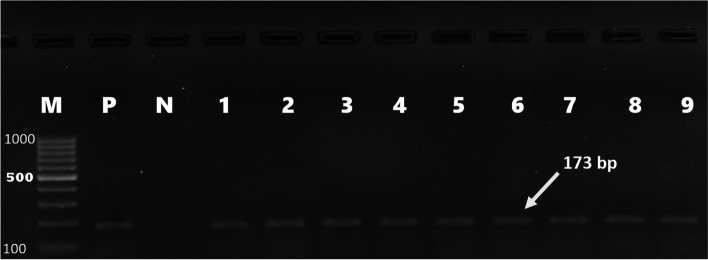
*tet(B)* gene detected using simplex PCR (M marker (Thermo Scientific, SM0241, 100 bp DNA Ladder), P positive control, N negative control, 1–9 positive isolates)

**Fig. 6:**
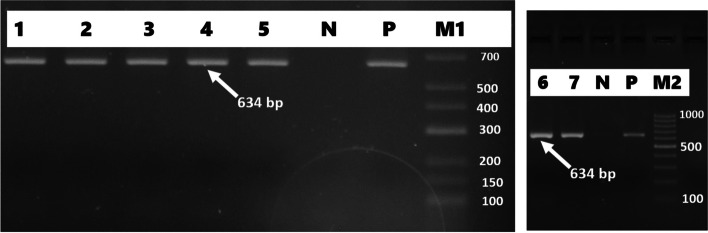
Determination of the *aphA1* gene causing kanamycin resistance by simplex PCR (M1 marker (Fermentas, SM1191, 100 bp DNA Ladder), M2 marker (Thermo Scientific, SM0241, 100 bp DNA Ladder) P positive control, N negative control, 1–7 positive isolates)

**Fig. 7:**
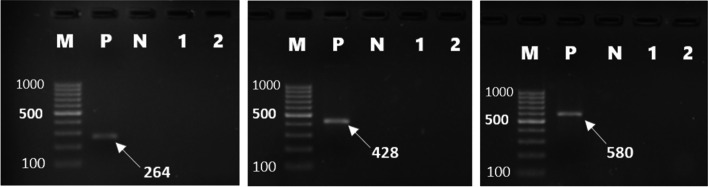
Determination of quinolone resistance genes (*qnrA* (580 bp), *qnrB* (264 bp), *and qnrS* (428 bp)) by PCR (*M* marker (Thermo Scientific, SM0241, 100 bp DNA Ladder), P positive control, N negative control, 1 and 2 negative isolates)

Forty-six (46%) of *E. coli* isolates were found to carry one or more of the sulfonamide resistance genes investigated. *sul1*(16%), *sul2* (26%), and *sul3* (34%) were detected in both gull and pigeon origin isolates (Figs. 2 and  3). Most sulfonamide resistance genes were found in combination. For example, *sul1*, *sul2,* and *sul3* (in 10 isolates), *sul2* and *sul3* (in 6 isolates), and *sul1* and *sul2* (in 4 isolates) genes were detected together. Comparing the two bird species *sul1* gene’s percentage was found to be significantly higher (*p* = 0.006) in gull isolates (26%) than in pigeon isolates (6%). In contrast, the percentage of the *sul3* gene was significantly (*p* < 0.001) higher in isolates of pigeon-origin (52%) (Table 4). Regarding the tetracycline resistance determinants, *tet(A)* and *tet(B)* were detected in 63% and 66% of the *E. coli* isolates (Figs. 4 and  5). The percentage of *tet(A)* gene was higher than *tet(B)* gene in pigeon isolates. On the other hand, the *tet(B)* gene percentage was higher than the *tet(A)* gene in gull isolates. Comparing the two bird species, a statistically significantly (*p* = 0.001) higher percentage of *tet(A)* gene was detected in the pigeon isolates. Among the aminoglycoside resistance determinants, *strA/strB* (24%) and *aphA1* (48%) were detected in both gull and pigeon isolates (Fig. 6). The percentage of *strA/strB* gene harborage in gull isolates (42%) was found to be significantly higher (*p* < 0.001) than in pigeon isolates. On the other hand, *tet(C)* gene, PMQR gene determinants (*qnr(A), qnr(B)* and *qnr(s)*), and some aminoglycoside modifying enzyme determinants *(aphA2, aadB* and *aac(3) IV)* were not found in any of the isolates. Figure 7 shows the negative results obtained after subjecting PCR amplicons to agarose gel electrophoresis to determine plasmid-mediated quinolone resistance determinants.

**Table 4:** Comparison of gulls and pigeons in terms of harboring *E. coli* isolates carrying antibiotic resistance genes

Drug group and resistance genes		The number of isolates carrying the resistance gene (%)		*χ*^2^	*P*-value
		Gull (*n* = 50)	Pigeon (*n* = 50)		
Tetracycline	*tet(A)*	23 (46)	40 (80)	12.39	< 0.001
	*tet(B)*	35 (70)	31 (62)	0.71	0.398
Aminoglycoside	*strA/strB*	21 (42)	3 (6)	17.76	< 0.001
	*aphA1*	18 (36)	30 (60)	5.76	0.016
Sulfonamides	*sul1*	13 (26)	3 (6)	7.44	0.006
	*sul2*	15 (30)	11 (22)	0.83	0.362
	*sul3*	8 (16)	26 (52)	14.44	0.000

### The association between phenotypic and genotypic resistance profile

Looking at the correlation between phenotypic and genotypic resistance profiles, 43.24% (16/37) of the streptomycin resistance isolates carried *strA/strB* gene, and the carriage of this gene was significantly (*p* = 0.001, *χ*^2^ = 11.92) associated with the streptomycin phenotypic resistance. Similarly, a significant association (*p* < 0.001, *χ*^2^ = 27.68) was found between *aphA1* gene carriage and kanamycin resistance, which was found in 81.57% (31/38) of kanamycin-resistant isolates. In addition, 9 *E. coli* isolates that showed intermediate phenotypic resistance to kanamycin were found to carry the *aphA1* gene. Thus, of the 48 *aphA1* genes identified, 31 were found in kanamycin-resistant isolates, while the others were found in isolates that were intermediately resistant (9) and susceptible (8) to kanamycin.

Regarding the relationship between tetracycline resistance and its genetic resistance determinants, *tet(A)* and *tet(B)* carriage was detected in 84.61% (44/52) and 76.92% (40/52) of tetracycline-resistant isolates, respectively. The carriage of these genes was also found to be significantly (*p* < 0.05) associated with tetracycline phenotypic resistance. All *E. coli* isolates phenotypically resistant to tetracycline had *tet(A)*, *tet(B),* or both. Moreover, 47.3% (9/19), 68.4% (13/19), and 36.8% (7/19) of the isolates phenotypically resistant to trimethoprim/sulfamethoxazole were found to carry *sul1*, *sul2,* and *sul3* genes, respectively. Despite the phenotypic resistance expression (e.g., fluoroquinolone and gentamicin), resistance genes were not detected in some *E. coli* isolates. On the other hand, some phenotypically susceptible isolates were also found to carry resistance genes (Table 5).

**Table 5:** The association between phenotypic and genotypic antibiotic resistance profiles of gulls and pigeon origin *E. coli* isolates

Drug group and resistance genes		Number of isolates carrying the resistance gene		*χ*^2^	*P*-value
		Resistant^a^	Non-resistant^b^		
Tetracycline	*tet(A)*	44 (84.61%)	19 (39.5%)	21.71	< 0.001
	*tet(B)*	40 (76.92%)	26 (54.16%)	5.76	0.016
Streptomycin	*strA/strB*	16 (43.24%)	8 (12.69%)	11.92	0.001
Kanamycin	*aphA1*	31 (83.73%)	17 (27.42%)	27.68	< 0.001

^a^Isolates that are phenotypically resistant to the indicated antibiotic and carry the resistance gene(s)

^b^Isolates phenotypically susceptible or intermediately resistant to the indicated antibiotic but carry the resistance gene(s)

### Genotyping of isolates by RAPD-PCR

**Fig. 8:**
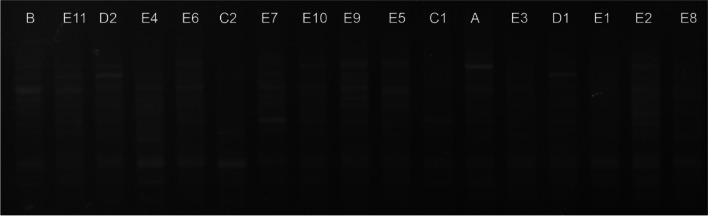
RAPD-PCR results of pigeon-origin *E. coli* strains

**Fig. 9:**
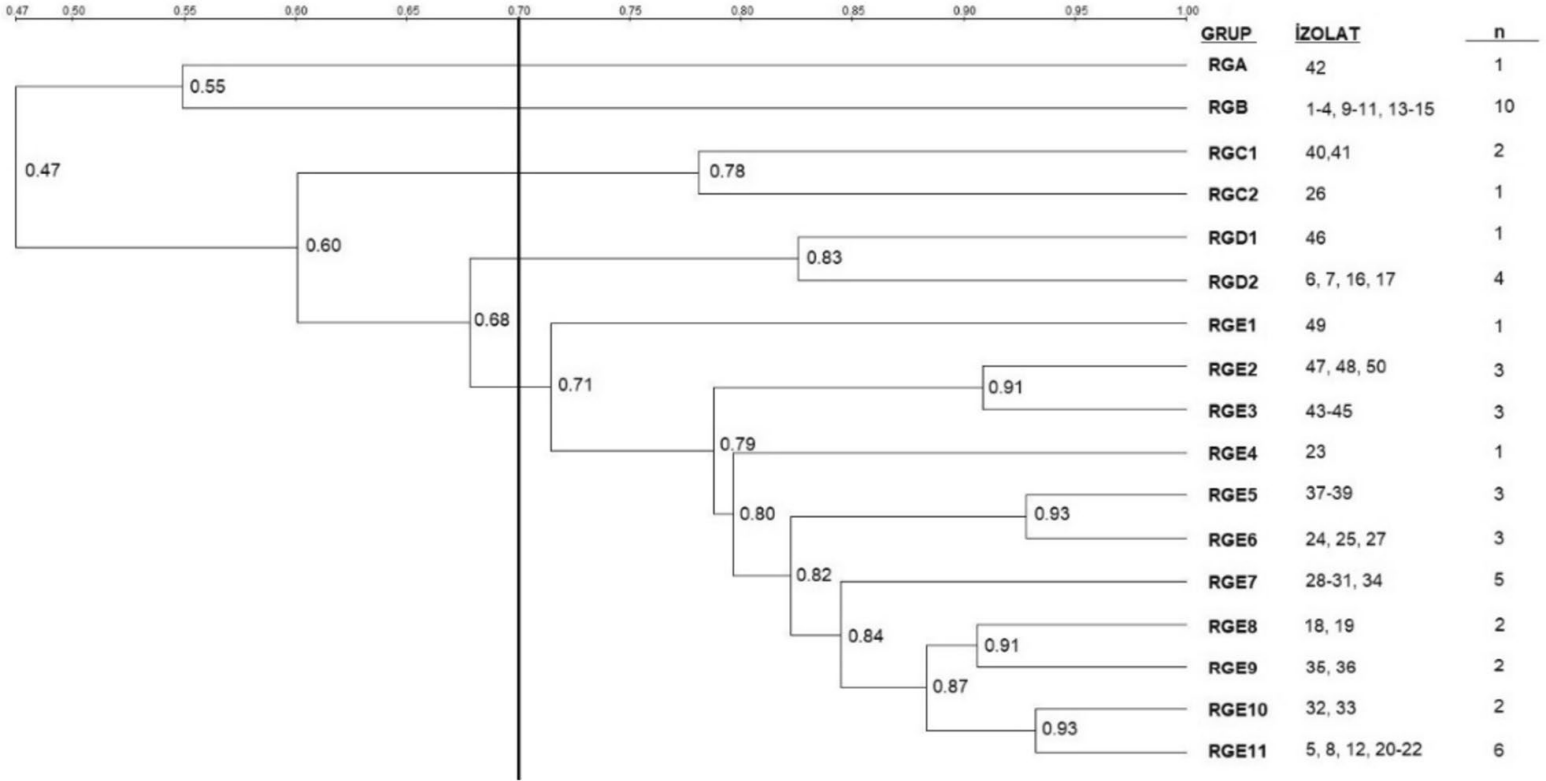
Phylogenetic analysis of pigeon origin *E. coli* strains

**Fig. 10:**
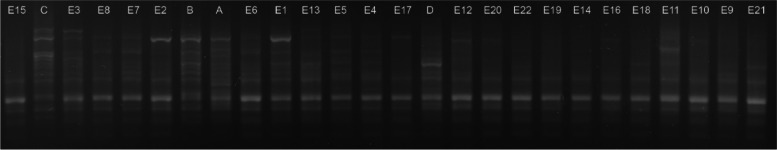
RAPD-PCR results of gull-origin *E. coli* strains

**Fig. 11:**
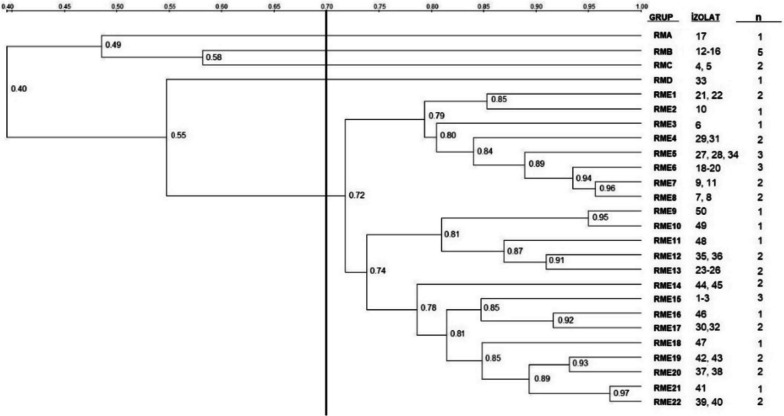
Phylogenetic analysis of gull origin *E. coli* strains

Dendrogram analysis revealed that the pigeon-origin *E. coli* strains had similarities ranging from 47 to 100%, and isolates’ genotyping based on a 70% similarity threshold resulted in two single (RGA, RGB) and three multiple (RGC, RGD, RGE) genotypes (Figs. 8 and 9). Among the multiple genotypes, RGC contained 3 isolates, RGD 5 and RGE 31 isolates. In the case of gull isolate, similarities ranging from 40 to 100% were found. Genotyping of gull isolates revealed four single (RMA, RMB, RMC, RMD) and one multiple (RME) genotypes based on the 70% similarity threshold. RME contained 41 isolates (Figs. 10 and  11).

## Discussion

Antibiotic resistance is a growing global challenge in both animal and human health that requires multisectoral action plans [12, 41]. A recent study by Murray et al. [14] attributed more than 1.2 million global deaths in 2019 to antimicrobial-resistant bacteria. Thus, microbial resistance to drugs of significant clinical importance is considered one of the biggest global public health threats challenging humans today [41], and it is estimated that if appropriate measures are not taken, its burden may increase and cause approximately 10 million deaths annually by 2050 [42]. Therefore, it is clear that a better understanding of antibiotic resistance situations, including resistance gene sources and vectors, is needed to reduce the potential risks associated with the increasing burden of antibiotic resistance [27, 31].

This study investigated the phenotypic and genotypic antibiotic resistance patterns of *E. coli* isolates isolated from gulls and pigeons. The results of the phenotypic antibiotic resistance test revealed higher antibiotic resistance to tetracycline (52%), kanamycin (38%), and streptomycin (37%). Of 100 investigated *E. coli* isolates, 63% were resistant to ≥ 1 antibiotic and 29% were found to be multidrug-resistant. A similar result was reported by Nowaczek et al. [52], who reported a 31.2% multidrug-resistant rate. Other researchers from Lithuania also investigated the resistance profile of *E. coli* obtained from various wild birds (including gulls) and reported a 33.5% MDR rate [17]. However, there are also studies reporting a high MDR rate ranging from 60 to 100% [3, 43–46] and lower MDR rates (below 20%) [47–51] from different parts of the world. These differences may be due to differences in bird species, other hosts with which they are in close contact, and geographical location.

Previous studies have reported a relatively high degree of resistance to tetracycline in wild bird-origin *E. coli* isolates from different countries [19, 25, 52–54]. Looking at the phenotypic resistance profiles of the isolates investigated in this study, a relatively higher degree of resistance to tetracycline (52%) was detected. This result was in agreement with recent studies reporting tetracycline resistance in *E. coli* isolates isolated from different wild bird species in Poland (50%), Australia (51%), Brazil (52.6%), and Italy (56%) [22, 25, 52, 55]. On the other hand, the rate of tetracycline resistance found in our study tended to be higher when compared with the results reported by Carroll et al. [50] (5.4%), Horn et al. [51] (10.91%), and Stedt et al. [28] (19%). However, other studies conducted in different parts of the world have reported high rates of tetracycline resistance, ranging from 80 to 100%, in a wide variety of wild bird species [18, 45, 46, 53]. The tetracycline group is of critical importance in the treatment of many infections in animals [12] and is also classified under “highly important antimicrobials” in the treatment of human infections [13]. The relatively high rate of tetracycline resistance detected the current study could be attributed to its widespread use in treating animal infections [56].

Resistance gene determination results revealed a significant association of *tet(A)* and *tet(B)* genes carriage with tetracycline resistance. Compared with *tet(B)*, the percentage of *tet(A)* gene’s carriage was found to be relatively higher in pigeon isolates, whereas the percentage of *tet(B)* gene was found to be higher in gull isolates. In overall results, unlike previous studies [18, 19, 57], the percentage of the *tet(B)* (66%) gene was found to be relatively higher than the *tet(A)* (63%). However, in agreement with our findings, other researchers have reported a higher percentage of *tet(B)* gene than *tet(A)* [37, 58]. Of tetracycline-resistant isolates, 84.61% and 76.92% carried *tet(A)* and *tet(B)*, respectively. In one study conducted in the Czech Republic, 55.1% (27/49) and 44.89% (22/49) of the black-headed gull (*Larus michahellis*) origin *E. coli* isolates resistant to tetracycline were reported to carry the *tet(A)* and *tet(B)* gene, respectively [48]. Another study by Merkeviciene et al. [17] reported that 85% and 18% of tetracycline-resistant *E. coli* isolates had *tet(A)* and *tet(B)* genes, respectively.

Looking at the aminoglycoside’s resistance profile, the streptomycin rate (37%) found in this study was comparable with a study from Australia that reported 34% resistance to this drug [59]. However, a higher resistance rate (ranging from 48 to 85%) [3, 43, 53–55] and a lower resistance rate (3% and 9.3%) [25, 48] have been reported in previous studies. Borges et al. (2017) reported 36.8% kanamycin resistance in Brazil, which agrees with a kanamycin resistance rate (38%) detected in this study. Moreover, the study by Barguigua et al. (2019) reported that 35% of *E. coli* isolates obtained from gulls were resistant to kanamycin. The kanamycin resistance rate found in this study was relatively higher when compared with the result of one study from Poland (18%) [52]. However, a higher kanamycin resistance rate (80.4%) has been reported in Turkey [30]. Among the aminoglycoside resistance genes investigated in this study, *aphA1* and *strA/strB* were found in 48% and 24% of the *E. coli* isolates, respectively. Moreover, it was determined that 81.57% of the kanamycin-resistant and 43.24% of the streptomycin-resistant *E. coli* isolates carried *aphA1* and *strA/strB* genes, respectively. Other researchers have also reported comparable results [17, 52].

The percentage of ampicillin-resistant isolates detected in the current study (28%) was in agreement with the result of Nowaczek et al. [52], who reported 28.1% resistance to ampicillin in wild bird origin *E. coli* isolates. Similarly, Hasan et al. [49] reported a 29.4% ampicillin resistance in *E. coli* isolates isolated from gulls (*Chroicocephalus brunnicephalus*) in Bangladesh. On the other hand, other researchers investigating the antibiotic resistance status in wild bird-origin *E. coli* isolates have reported a higher ampicillin resistance ranging from 70 to 100% [3, 53, 55, 60] and lower ampicillin resistance rate (less than 20%) [19, 48].

A recent study from Malaysia reported 16.7% trimethoprim/sulfamethoxazole resistance, which is consistent with the finding of this study (19%). A relatively lower trimethoprim/sulfamethoxazole resistance rate was found in pigeon-origin isolates (6%) compared to gull isolates (32%). Comparable findings were reported from Brazil (3.9% in pigeon origin *E. coli* isolates) [61] and Italy (26.6% in gull origin *E. coli* isolates) [25]. *sul1*, *sul2*, and *sul3* genes, which cause sulfonamide resistance, were detected in isolates isolated from gulls and pigeons. Other studies conducted in China [19] and Lithuania [17] have also reported the commonness of these genes in *E. coli* isolates isolated from wild birds. Furthermore, 96% of trimethoprim/sulfamethoxazole-resistant *E. coli* isolates were found to carry one or more *sul* genes. Comparable results were reported in a study from Portugal and Slovakia [54, 62].

Regarding the chloramphenicol resistance, 21% of the isolates were found to be resistant to this antibiotic, with 24% of the gull-origin isolates and 18% of the pigeon-origin isolates being chloramphenicol resistant. WHO classified chloramphenicol under “highly important antimicrobials” in human health [13]. The use of chloramphenicol in food animals is prohibited in many countries [56], including Turkey [63]. In this case, less exposure of wild birds to this antibiotic is expected due to lower levels of antibiotic residues in the environment [64]. Despite this, resistance to this antibiotic has been reported in *E. coli* isolates isolated from various animal species, including cattle [56, 65]. The possible reason for this could be the persistence of antibiotic-resistant bacteria in the environment [66]. Even though the result obtained in this study tends to be higher when compared with the result of the study from Italy (0%) [25], Czech Republic (1.9%) [48], Bangladesh (2.4%) [49], Poland (6.25%) [52], and China (11.1%) [19], a comparable finding has been reported from Singapore (19.2%) [64]. Moreover, higher resistance to this antibiotic has been reported in Bangladesh (43.64%) [3] and Portugal (41.7%) [54].

Gentamicin and ciprofloxacin are antibiotics of critical importance in treating human infections [13]. Resistance to fluoroquinolones (12%) and to gentamicin (13%) was detected at a lower rate. Enrofloxacin, gentamicin, and ciprofloxacin resistance were not detected in any of the pigeon-origin *E. coli* isolates. Interestingly, these results were found to be consistent with one study that analyzed the antibiotic susceptibility profile of *E. coli* isolates obtained from domestic pigeons (*Columba livia domestica*) in Turkey and reported 100% susceptibility to enrofloxacin and gentamicin [67]. Furthermore, Silva et al. [62] reported 100% gentamicin susceptibility of pigeon-origin *E. coli* isolates, which is consistent with the findings of this study. Ong et al. [65] reported 11.5% resistance against ciprofloxacin. Russo et al. [25] also reported 13.3% and 16.6% resistance to gentamicin and enrofloxacin, respectively. In contrast to this study’s findings, one study from Egypt reported resistance to ciprofloxacin and gentamicin at 88% and 80%, respectively [43].

Although a statistically significant association between phenotypic and genotypic antibiotic resistance was found in most cases, isolates showing phenotypic resistance without corresponding resistance genes and isolating carrying resistance genes without phenotypic resistance were also encountered. For example, all tetracycline-resistant isolates were found to carry *tet(A)*, *tet(B)*, or both and harboring these genes was found to be significantly (*p* < 0.05) associated with tetracycline phenotypic resistance. Similar results were obtained during the analysis of the association between streptomycin resistance and *strA/strB* gene carriage and kanamycin resistance and harborage of the *aphA1* gene. On the other hand, despite the isolates’ phenotypic resistance to fluoroquinolones and gentamicin, the genes responsible for the resistance of these antibiotics could not be detected in any of the resistant isolates, which indicates as phenotypic and genotypic antibiotic resistance may not always be consistent. The same situation has been reported in previous studies [68, 69].

The possible explanation for the detection of antibiotic resistance genes in isolates without phenotypic antibiotic resistance is that the detected genes may not be expressed or non-functional [68, 70]. On the other hand, the absence of investigated resistance genes in phenotypically resistant isolates could be explained by the presence of alternative resistance mechanisms other than those investigated. For example, *E. coli* can develop resistance to fluoroquinolones through chromosomal mutations of *gyrA* and *parC* genes, which encode quinolone targets (DNA gyrase and topoisomerase). This resistance mechanism is vertically transmitted and is commonly known to confer high levels of resistance to quinolones, in contrast to horizontally transmitted plasmid-mediated quinolone resistance (like *qnr*) that confers low levels of quinolone resistance [71, 72].

RAPD-PCR is an effective method to assess the genetic diversity of many bacterial species [73] and is also being used in evaluating the genotypic relationships of wild bird origin *E. coli* isolates [32, 74]. Although the origin or source of the pathogen was not determined in this study, phylogenetic analysis based on RAPD-PCR results revealed genetic similarity ranging from 47 to 100% in pigeon isolates and 40 to 100% in gull isolates. The strains with high similarities (> 90%) may have been acquired from the same sources. However, this needs to be elucidated using more advanced typing techniques.

Antibiotic-resistant bacteria in wild birds are often associated with environmental contamination from animal and human wastes rather than direct exposure to antibiotics [21, 75, 76]. In particular, the level of anthropogenic impact (e.g., wastewater, waste from livestock farms, landfill) in a given area can affect the likelihood of wild birds becoming infected by antibiotic-resistant bacteria [77]. Factors such as urbanization and the loss of natural wildlife habitats, which increases the contact of birds with contaminated environments, aggravate this situation by facilitating the infection of birds with antibiotic-resistant bacteria [22]. Thus, the difference in antibiotic resistance percentage detected in this study and other studies may be attributed to the level of access of wild animals to anthropogenic sources, which may vary from country to country and even in different places in the same country, and bird’s foraging strategies that may differ according to bird species [78]. On the other hand, the low resistance percentage detected in this study (enrofloxacin, ciprofloxacin, and gentamicin resistance) and the susceptibility of all isolates to cephalothin may indicate that these bird species had less or no contact with environments contaminated with bacteria resistant to these antibiotics.

## Conclusions

This study revealed that wild birds (gulls and pigeons) carry strains of *E. coli* that are resistant to even drugs of critical importance in human health. In addition, the isolates were found to carry resistance genes like *tet (A)* and *tet(B)*, *strA/strB*, *aphA1*, *sul1*, *sul2,* and *sul3*, which provide resistance to drugs that have an essential role in human and veterinary medicine. These results suggest that wild birds may serve as a reservoir for multidrug-resistant bacteria and resistance genes. Given the increasing threat of antibiotic resistance in both human and animal health, the detection of MDR bacteria carrying antibiotic resistance genes from free-living wild animals deserve more attention. Specially gulls and pigeons, which are in close contact with humans, may pose a public health risk by contributing to the spread of resistant bacteria through fecal contamination of the environment. Therefore, multisectoral collaboration under the “One Health” umbrella is of critical importance in addressing antibiotic resistance challenges. Continuous monitoring of the possible antibiotic resistance reservoirs, appropriate drug prescription, awareness creation, and searching for alternative treatment options is paramount. Although this study has identified the carriage of multidrug resistance with resistance genes in *E. coli* isolates of gull and pigeon origin, further molecular studies are warranted to elucidate the source of infection for wild birds and possible interspecies transmission of resistant bacteria.

## Data Availability

The data that support the findings of this study are available from the corresponding author on reasonable request.
